# Objective Neighborhood-Level Disorder Versus Subjective Safety as Predictors of HIV Transmission Risk and Momentary Well-Being

**DOI:** 10.1007/s10461-024-04413-z

**Published:** 2024-07-04

**Authors:** Leigh V. Panlilio, Kenzie L. Preston, Jeremiah W. Bertz, Landhing M. Moran, Matthew Tyburski, Sara K. Hertzel, Shireen Husami, Fatumastar Adan, David H. Epstein, Karran A. Phillips

**Affiliations:** 1 Intramural Research Program, National Institute on Drug Abuse, Baltimore, MD USA; 2 National Institutes of Health, NIDA Intramural Research Program, Translational Addiction Medicine Branch (TAMB), RAPT (Real-World Assessment, Prediction, and Treatment) Unit, 251 Bayview Blvd., Suite 200, Baltimore, MD 21224 USA

**Keywords:** HIV, Substance use disorders, Behavioral geography, Activity space

## Abstract

Mental health and HIV risk behavior have been studied with ecological momentary assessment (EMA), but this approach has not been combined with tracking of activity space (where people go and what they encounter there) in people with HIV and their social relations, who may be HIV+ or HIV−. Activity space represents a modifiable risk or protective factor for behavior related to health status and quality of life, in both clinical and nonclinical populations. We conducted an observational study with 286 participants (243 HIV+ and 43 HIV−), roughly matched for socioeconomic status and neighborhood of residence via three waves of snowball sampling. Each participant carried a smartphone for up to 4 weeks, making 5 randomly prompted entries and 1 end-of-day entry each day, plus self-initiated event-contingent entries for sexual activity and drug use. Responses to randomly prompted items provided subjective evaluations of the safety of the participant’s current social and physical environment (the place they were and the people they were with). GPS-based location tracking—coupled with publicly available statistic indicating neighborhood-level physical disorder and socioeconomic disadvantage—provided an indicator of each participant’s exposure to objective psychosocial hazard. We examined possible relationships of these objective and subjective environmental exposures with risky sexual and intravenous drug-use behavior, knowledge and utilization of antiretroviral treatment and prophylaxis, and momentary mental health (mood and stress, which relate to risky behavior and overall well-being). We found that both risky behavior and mental health were more related to participants’ subjective evaluations of their activity space than to objective measures of neighborhood-level disorder, suggesting that, even within an objectively hazardous neighborhood, people who find a niche they perceive as socially and physically safe may engage in less risky behavior and have better well-being.

*Trial registration* Clinicaltrials.gov Identifier NCT01571752.

**Supplementary Information:**

The online version contains supplementary material available at 10.1007/s10461-024-04413-z.

## Introduction

Health problems are more likely for people who live in neighborhoods that are physically disordered and socioeconomically disadvantaged (i.e., places with high rates of crime, social disorganization, physical disorder, and low socioeconomic status) [1, 2]. Residents of such neighborhoods tend to have higher rates of HIV transmission-risk behavior and higher rates of HIV infection [3–6]. HIV-positive (HIV+) people who live in disordered neighborhoods tend to have later initiation of treatment and worse health outcomes compared to those in other neighborhoods [7–9].

Along with these general relationships between neighborhood disorder and poor health outcomes, there is variability in outcomes between individuals who live in disordered neighborhoods, with some faring better than others. We have previously found that—on a within-person level—many residents of disordered neighborhoods feel more comfortable and less stressed while they are in a neighborhood that is more disordered, by objective measures such as crime levels and number of abandoned houses [10]. This somewhat paradoxical finding shows that people’s perceptions and evaluations of their environment do not always coincide with more objective evaluations, and it suggests that—within a physically and socioeconomically disordered neighborhood—many people have a niche, a microenvironment where they are safe and have social ties. Thus, the relationship between health and environment is largely determined by general, objective properties of a person’s neighborhood, but it can also be influenced by the person’s subjective evaluation of the specific places where they spend time and the people they interact with there.

In previous studies, we used geographical momentary assessment (GMA)—i.e., ecological momentary assessment [11] (EMA) combined with Global Positioning System (GPS) tracking—to study mood and behavior across groups that differed with respect to neighborhood characteristics [10], hepatitis C status [12], and drug-use patterns [13]. In the present study, we used GMA to study HIV transmission-risk behavior and momentary psychological well-being in a sample of HIV+ individuals and their close social companions who are HIV−.

In previous studies by others [14, 15], retrospective interviews provided information on the types of locations where HIV transmission is more likely. Our approach extends the interview-based retrospective approach used by Mason and colleagues [16–18] to characterize activity space based on: (1) participants’ subjective descriptions of their current environment, combined with (2) GPS-based assessment of exposure to objective neighborhood-level characteristics (e.g., socioeconomic status, crime levels, physically disordered infrastructure) that are known to be associated with public health [1, 2]. Specifically, we designed this study to gain insight into the relationships between activity space (the set of locations through which people move in their daily lives [19]) and HIV transmission-risk behavior in HIV+ individuals and their social relations. We also sought to determine whether activity space is associated with utilization of HIV-related healthcare (antiretroviral medication) and with mental-health variables (stress and mood) that are (1) often problematic for people who are HIV+ [20, 21] and (2) might increase risky sexual behavior and intravenous drug use [22–25]. Ultimately, information about these relationships between environment, mental health, and behavior could be useful for reducing transmission of HIV and other infections, increasing entry into prevention and treatment services, and reducing health disparities in rates of new infections and in treatment outcomes for people living with HIV.

## Methods

### Overview

To understand relationships between environment and behavior in HIV+ people and HIV− people within their social network, we conducted GMA over a 4-week period in city dwellers within reach of our research clinic. Participants were prompted at random times each day to categorize their current environment by describing the place they were and the people who were there, and to rate their current levels of positive mood, negative mood, and stress. Participants also provided event-contingent reports describing potentially risky behaviors (sexual activity and intravenous drug use) when they occurred. We assessed each participant’s exposure to neighborhood-level disorder by matching GPS tracking data to publicly available statistics [1, 2].

### Recruitment

We first recruited HIV+ adults (Wave 1) and asked them each to recruit, from within their close social network, three more participants (Wave 2), who could be HIV+ or HIV−. Wave 2 participants who were HIV+ were asked to recruit 3 more participants (Wave 3) from within their network, who could be HIV+ or HIV−. We continued this recruiting strategy until we reached a target of 300 consenting participants (a sample-size choice driven largely by available resources). Similar respondent-driven sampling procedures have been used previously to study hidden populations (i.e., groups that might be reluctant to disclose themselves) [26, 27].

### Data Collection

#### Treatment Compliance and PrEP Knowledge

The HIV-Risk Timeline Follow-Back Interview [28], which was administered during the initial study visit (prior to the EMA portion of the study) included an item asking participants who had a prescription for antiretroviral medication how long it had been since they had missed a dose; there were six possible answers, constituting an ordinal scale: “Never,” “More than 3 months ago,” “1–3 months ago,” “2–4 weeks ago,” and “within the past week.” Familiarity with pre-exposure prophylaxis (PrEP) was assessed with an item from the HIV History questionnaire [29]; the possible answers were “Not at all familiar,” “Familiar, but I’ve never received PrEP,” and “Very familiar (I receive or have received PrEP).” Note that these two variables were assessed prior to the collection of GMA data, but we refer to them as outcomes based on the assumption that the GMA-based predictor variables mostly reflect stable characteristics of the participants and their environments that were not altered by being in the study.

#### Geographical Ecological Momentary Assessment

Each participant carried a study-issued smartphone for up to 4 weeks. Location data was recorded once every 5 min, whenever the phone was powered on and receiving a GPS signal. We prompted participants to make five randomly prompted (RP) reports per day and one prompted end-of-day (EOD) report per day, and in addition to these RP and EOD reports, we asked them to make ad hoc self-initiated reports of sexual activity or drug use (event-contingent reports, described below). The randomly prompted items asked for subjective descriptions of the current environment, concerning the place and the people there, and ratings of the participant’s current mood and level of stress.

#### Measures of Risky Sexual and Drug-Use Behavior (Event-Contingent EMA)

Participants initiated event-contingent reports when they engaged in sexual activities with a partner or used drugs. These reports provided information about transmission risk, including condom use, having sex for money, drugs or things, having sex with a partner who has sex for money, drugs or things, having sex with a casual partner, having sex with a partner who uses IV drugs, and injecting drugs using “works” that had been used by another person (needle sharing). For analysis, each of these risky behaviors was coded as a binary outcome, indicating whether the participant reported engaging in the behavior at all during the study.

### Data Analysis

#### Strategy

We assessed three kinds of outcomes: (1) behaviors that carry risk for HIV transmission; (2) knowledge of and compliance with HIV-related healthcare; and (3) momentary psychological well-being. We modeled these outcomes as a function of two composite measures of activity space: (1) neighborhood-level psychosocial hazard scores and (2) participants’ evaluations of the place they were and the people they were with when they were randomly prompted to provide an EMA report. Based on the sources of the information used to construct these composite measures (publicly available statistics vs. self-reported EMA ratings) and based on how the information was encoded (as neighborhood disorder vs. perceived positive qualities of the environment), we refer to the composite measures as “objective hazard” and “subjective safety,” respectively.

#### Objective Hazard Scores

To characterize activity spaces by publicly available information related to neighborhood-level disorder and socioeconomic status, we used psychosocial-hazard scores that were designed to assess “stable and visible features of neighborhood environments that give rise to a heightened state of vigilance, alarm, or fear in residents” [1] and that “may be an important link between neighborhood socioeconomic disadvantage and adverse health outcomes.” [2]. These scores were calculated based on neighborhood statistics in four domains: social disorganization, public safety, physical disorder, and economic deprivation. Data for these domains were obtained from the Baltimore Neighborhood Indicator Alliance (BNIA; https://data-bniajfi.opendata.arcgis.com). Specifically, for each neighborhood in each year, psychosocial-hazard scores were derived from the percentage of households that were headed by women with children under 18, percentage of population less than 26 years old with less than high-school or equivalent education, rates of shootings, rates of violent crime, percentage of properties vacant or abandoned, liquor store density, household income (reverse scored), percentage of households below the poverty line, rates of unemployment, and rates of certain types of calls for service (domestic violence; common assault; narcotics; automobile accidents; fire and EMS; total 911 calls; and complaints about street conditions, including dirty streets and alleys, clogged storm drains, or streetlight outages). Each of these individual measures was standardized to have a mean of 0 and standard deviation of 1, and then averaged within each domain, then averaged across domains to provide an overall psychosocial hazard score for each neighborhood in Baltimore city during each year of the study (2017–2019). Neighborhoods were defined by the GPS boundaries of the BNIA Community Statistical Areas. Hazard scores were not available for areas outside of Baltimore City, but all participants spent all or most of their time within city limits. For analysis, we used GPS to identify the neighborhood the participant was in when each randomly prompted report was completed, then averaged these values to calculate the mean hazard score within each participant, referred to below as the raw hazard score, with zero representing the average for all neighborhoods in the city and units representing standard deviations of the mean. For use as a regressor in statistical models, the raw hazard scores were standardized across all participants to obtain personal hazard scores, representing each participant’s personal level of exposure to neighborhood-level psychosocial hazard during the study, relative to the other participants, with zero representing the sample mean.

#### Subjective Safety Scores

We used participants’ responses to random-prompt EMA items to quantify their activity space in terms of their personal descriptions of the place they were (“The place where I am now is…”) and who they were with (“The people I am with now are…”). These items were loosely based on items from the Perceived Neighborhood Scale (PNS) [30], adapted for EMA by asking about the participant’s current environment rather than the original instrument’s focus on “the neighborhood you live in.” All items used to calculate subjective safety scores were rated on a 1–5 Likert scale and expressed (with reversed scores when appropriate) to reflect positive qualities. Subjective safety scores were calculated for each participant as the mean of three equally weighted components: (1) mean endorsement of the items describing the current place as “safe,” “comfortable,” and “a place where I belong”; (2) endorsement of the single item describing the current place as “a place I would rather not be,” reverse scored; and (3) mean endorsement of the items describing “the person or people I am with” as: “a good influence,” “cares about me,” “would help me,” “is in my close social circle,” “I enjoy being around,” “I feel safe around,” “I hang out with,” and three reverse-scored items, “makes me feel like using alcohol or drugs,” “I am angry with,” and “I would rather be with someone else.” Objective hazard scores and subjective safety scores were each calculated as a personal average for each participant, to reflect their level of exposure during the study. These hazard and safety scores were only weakly correlated with each other (r = − 0.095, p = 0.11).

#### Stress and Mood Outcomes (Mental Health)

Stress was assessed based on a single item from random-prompt reports, “How much stress are you feeling right now?,” which used a 1–5 Likert scale, with 5 indicating more stress. Positive mood and negative mood scores were based on endorsements of items in a list of mood adjectives that the participants rated using a Likert 1–5 scale in each random prompt report, in response to the question, “How do you feel right now?” Based on nonmetric dimensional scaling of the mood items, we calculated a personal-average positive mood score (median of contented, relaxed, pleased, happy, cheerful, and lively) and negative mood score (median of bored/lonely, afraid, annoyed/angry, sad, uneasy, exhausted, stressed, anxious, and overwhelmed) for each participant, averaged across the study.

#### Statistical Modeling

The objective hazard and subjective safety regressors were each standardized and used in a model for each outcome using R [31] with Bayesian Regression Models using Stan (brms [32] package, version 2.17.0) with weakly regularizing priors and generalized linear model families: Bernoulli (for the risky behavior outcomes, which were binary, indicating whether the participant had engaged in the behavior during the study) and cumulative probit (for ordinal outcomes, based on Likert ratings or the scales used to assess PrEP familiarity and time since missing a dose of antiretroviral medication).

#### Assessment of Regression Results

We used the posterior distributions from the models to plot the estimated regression coefficients and conditional effects, and to obtain Bayesian p values using the pd_to_p function from bayestestR [33]. Plots of posterior distributions of regression coefficients are more informative than p-values because they fully represent uncertainty and the most likely values of the parameter. Results in tables and figures are presented separately for binary and ordinal outcomes. Regression coefficients (reported as “b” in the text) are plotted as odds ratios for binary outcomes and as standardized regression coefficients for ordinal outcomes. Conditional effects (the effect of each regressor with the other regressor held at its mean) are plotted with 90% credible intervals. Numeric results for all regressions are provided in the supplementary tables.

#### Hypotheses

Based on the behavioral-geography literature showing that neighborhood disorder is generally associated with worse health outcomes, we expected participants with more exposure to disordered neighborhoods (i.e., participants with higher objective hazard scores) to have poorer adherence to antiretroviral medication schedules, lower familiarity with PrEP, and higher probability of behavior that could lead to HIV transmission (risky sexual behavior, needle sharing). Based on previous findings that personal evaluations of one’s environment are important but do not always agree with objective measures of environmental hazard, we expected higher subjective safety scores to be associated with effects opposite to those expected for objective hazard (i.e., that subjective safety would have protective effects). Note that the opposing directions of the two effects reflect our arbitrary choice to code objective hazard and subjective safety rather than coding both as hazard (or both as safety). Regardless of these coding choices, we expected both the objective and subjective measures to be associated with the outcomes.

## Results

### Recruiting and Networks

The types of networks obtained through the recruiting procedure are shown in Supplementary Fig. S1. Of the 172 participants from Wave 1, 53 recruited at least one other participant. Overall, there were 131 participants who had a network within the study. Most of the networks consisted of two participants (one from Wave 1 and one from Wave 2), and about a third of the networks consisted of more than 2 participants. There were 17 HIV+ participants who recruited one other HIV+ participant (i.e., 12% of participants were in a dyad with no HIV− members), and there were 159 HIV+ participants (56% of the total sample) who recruited no other participants. Overall, there were more HIV+ participants than HIV- participants in the study (5.6:1 ratio; see Table 1 for demographics). Given that all participants were either HIV+ or a close social relation of a participant who was HIV+, the behaviors we assessed could carry risk of transmission either to or from the participant, so HIV+ and HIV− participants were included together in all models. The overall sample was predominantly Black, and the average age was about 50. The percentage of participants who were male was 1.8 times higher in the HIV+ group than in the HIV− group. Of the 300 participants recruited, 286 participants provided GMA data.

**Table 1:** Demographics by HIV status

	HIV+ (N = 243)	HIV− (N = 43)	Overall (N = 286)
Age			
Mean (SD)	51.4 (10.1)	46.2 (12.8)	50.6 (10.6)
Median [min, max]	54.0 [19.0, 70.0]	48.0 [25.0, 69.0]	53.0 [19.0, 70.0]
Gender			
Female	82 (33.7%)	28 (65.1%)	110 (38.5%)
Male	157 (64.6%)	15 (34.9%)	172 (60.1%)
Transgender	4 (1.6%)	0 (0%)	4 (1.4%)
Race			
Black	208 (85.6%)	32 (74.4%)	240 (83.9%)
Multi	11 (4.5%)	0 (0%)	11 (3.8%)
Unknown	6 (2.5%)	3 (7.0%)	9 (3.1%)
White	18 (7.4%)	8 (18.6%)	26 (9.1%)
Orientation			
Asexual	2 (0.8%)	2 (4.7%)	4 (1.4%)
Bisexual	34 (14.0%)	8 (18.6%)	42 (14.7%)
Gay	60 (24.7%)	5 (11.6%)	65 (22.7%)
Heterosexual	134 (55.1%)	24 (55.8%)	158 (55.2%)
Other	13 (5.3%)	4 (9.3%)	17 (5.9%)

### Raw Hazard and Safety Scores

In the rest of this paper, we present results based on hazard and safety scores standardized to have a mean of zero and standard deviation of 1, to assess differences between participants in the study relative to each other. Within this section, however, we characterize the raw objective hazard scores, because they describe the neighborhoods where participants spent time during the study, relative to the city as a whole. There was a wide range of raw objective hazard scores, but on average, participants spent time in neighborhoods with scores that were higher than the mean for all neighborhoods in the city (zero). Specifically, the raw objective hazard scores in our sample had a mean of 3.23, standard deviation (sd) of 4.55, median of 3.75, and interquartile range (iqr) of 6.38. The percentage of participants whose raw hazard scores were above zero was 75.9, and the percentage whose scores were below zero was 24.1. Raw values of the subjective safety scores can be compared to the range of the scale prior to standardizing (scored from 1–5 with higher indicating stronger endorsement). Average raw values for the three components of the subjective-safety regressor were: Place I am safe/comfortable/belong (mean = 3.97, sd = 0.84, median = 4.09, iqr = 1.28), Place I’d rather not be (mean = 1.7, sd = 0.74, median = 1.43, iqr = 0.97); People I am with (scored to be higher if positive, mean = 3.7, sd = 0.72, median = 3.73, iqr = 1.12). These values indicate that most participants were in the upper range of possible scores for the positive components and in the lower range for the negative components. That is, most participants felt subjectively safe most of the time. All considerations of objective hazard and subjective safety scores in the text below this point refer to the non-raw scores, standardized within the sample to reflect effects relative to other participants.

### Transmission-Risk Behavior

**Fig. 1:**
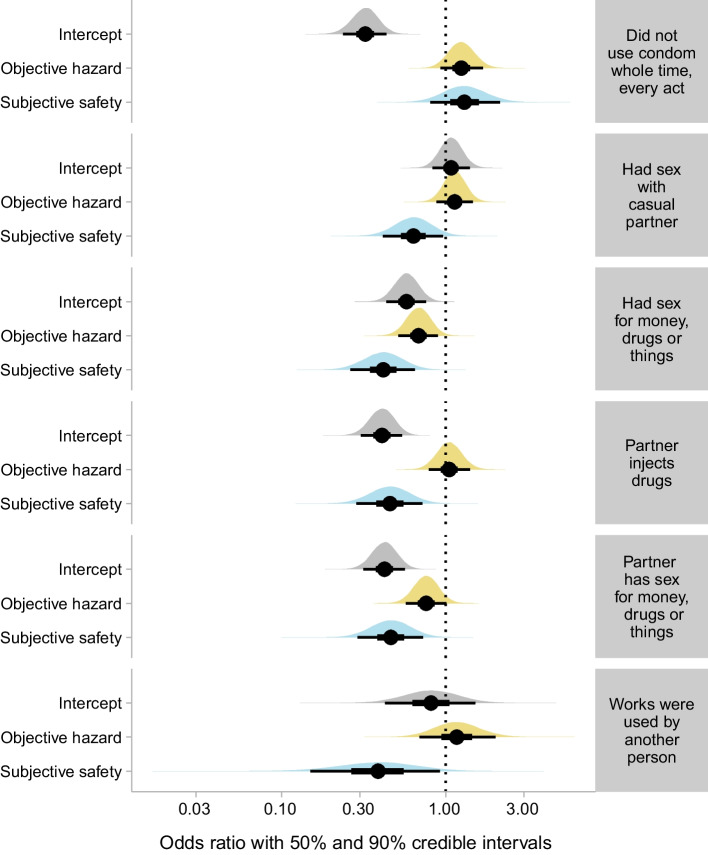
Odds ratios for the intercept, objective hazard regressor, and subjective safety regressor from each model of a transmission-risk behavior treated as a binary outcome (i.e., whether a participant engaged in the behavior at all during the study). All coefficients are expressed as odds ratios (obtained by exponentiating the model coefficients). Intercepts indicate the odds of the outcome when both predictors (objective hazard in yellow and subjective safety in blue) are at their mean (0). Odds ratios for the predictors indicate the change in odds associated with a change of one standard deviation in the predictor. Each outcome (identified by text in gray boxes) was modeled separately. Circles indicate the mean estimate. Thick confidence bars indicate the 50% credible interval, and thin confidence bars indicate the 90% credible interval. Densities illustrate the whole posterior distribution for each coefficient. Predictors are standardized, with 0 indicating the mean across all participants

**Fig. 2:**
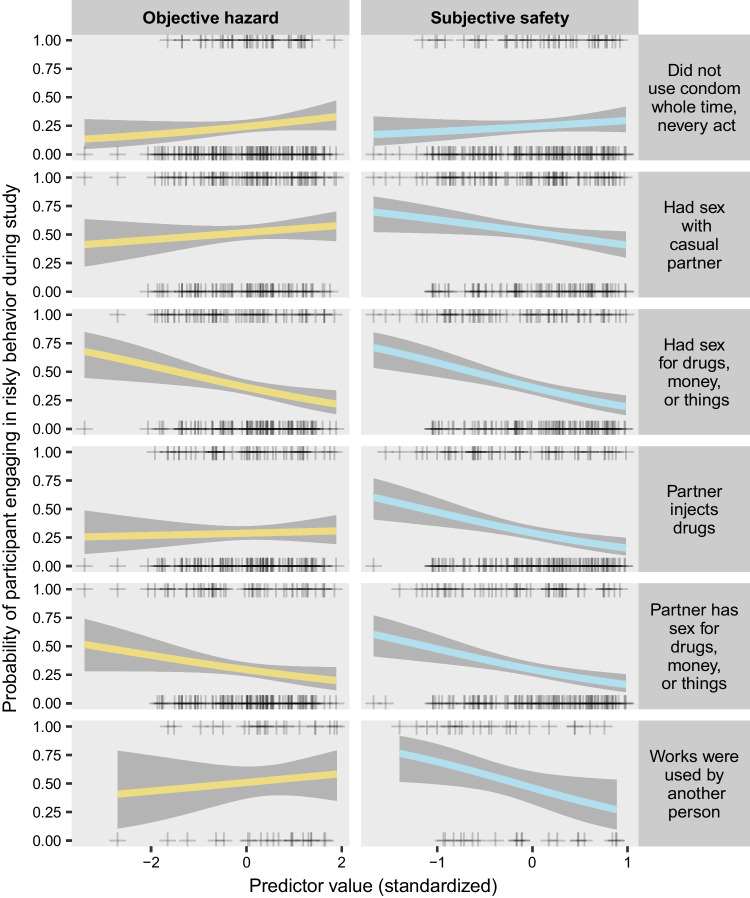
Illustration of the estimated probability of each transmission-risk behavior as a function of each predictor, from the same models shown in Fig. 1. Each plus sign shows the observation for a single participant, with a value of 1 on the y-axis indicating that the behavior occurred at least once during the study, and 0 on the y-axis indicating that it did not occur. Colored lines indicate the median of the posterior distribution, and gray confidence bands indicate the 90% credible interval

We expected exposure to disordered neighborhoods to be associated with higher probability of risky behavior, but we found no evidence for this (see Figs. 1 and 2; coefficient tables for all models are provided in Supplementary Table S1). Among the transmission-risk outcomes, objective hazard only had a reliable association with having sex for drugs, money, or things, and this behavior was *less* likely in participants who were exposed to neighborhoods with higher hazard scores (b = − 0.38, p = 0.0212). To a lesser extent, but in the same direction, having sex with a *partner* who has sex for money, drugs or things was also less likely in participants with higher objective hazard scores (b = − 0.28, p = 0.1108). Unlike objective hazard, subjective safety *was* associated with many of the outcomes in the direction we expected. Specifically, higher subjective safety was associated with lower likelihood of engaging in 5 of the 6 risky behaviors we assessed: having sex for money (b = 0.28, p = 0.0007), having sex with a partner who has sex for money (b = − 0.77, p = 0.0045), having sex with a partner who injects drugs (b = − 0.79, p = 0.0051), sharing needles (in the 42 participants who reported IV drug use during the study; b = − 0.97, p = 0.0709), and having sex with a casual partner (b = − 0.46, p = 0.072).

### Treatment Adherence

**Fig. 3:**
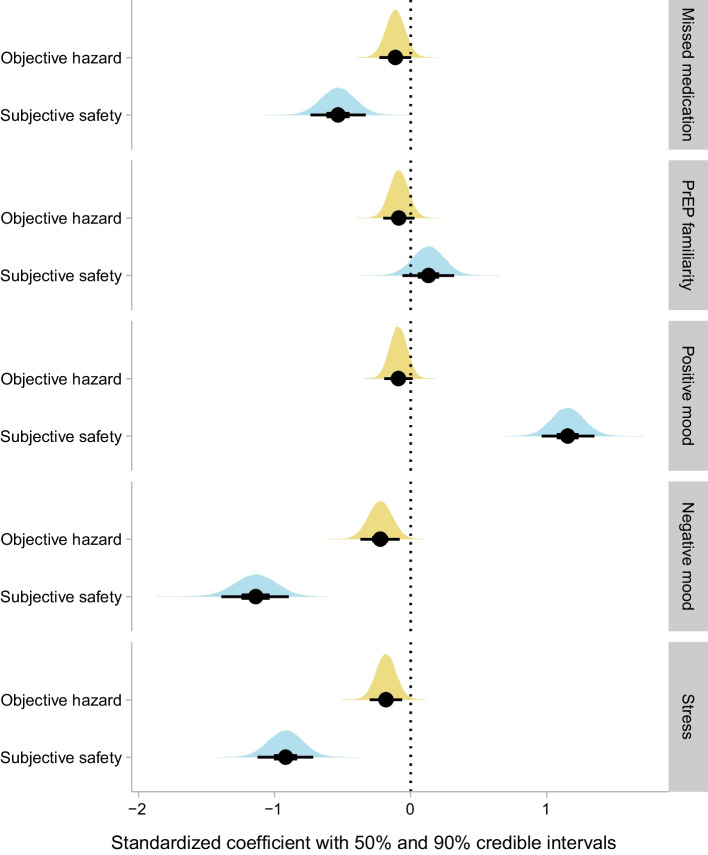
Standardized coefficients for the objective hazard regressor and subjective safety regressor from each model with an ordinal outcome. Details are the same as for Fig. 1 except that the results are from cumulative probit models, and the results are expressed as standardized coefficients rather than odds ratios

**Fig. 4:**
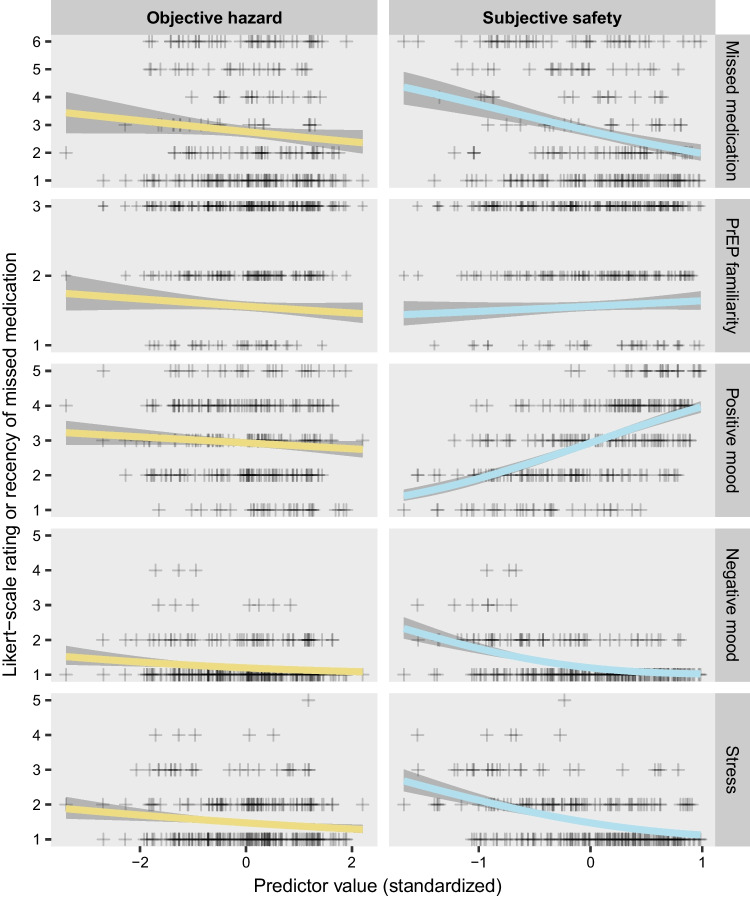
Illustration of the relationship between the regressors (objective hazard and subjective safety) and each ordinal outcome, from the same models shown in Fig. 3. Each plus sign shows the personal median observation for a single participant, on the ordinal scale. Colored lines indicate the median of the posterior distribution, and gray confidence bands indicate the 90% credible interval. Note that negative mood had 5 possible levels, but no participant’s median was higher than 4. Textual descriptions of the levels for each ordinal outcome are provided in the “Data collection” section of the text. For simplicity of presentation, this figure depicts the y-axis as continuous, ignoring the thresholds that are a part of the model. A more detailed figure, taking the thresholds into account and showing the estimated probability of each level of the ordinal outcome (but without graphic representation of the individual observations), is presented in Supplementary Fig. S2

In the 247 participants who had a prescription for antiretroviral therapy (Figs. 3, 4), those who had higher levels of subjective safety were much more likely to report never having missed a dose of medication (b = − 0.53, p < 0001) (Supplementary Figure S2). In a weaker effect that was contrary to expectations, higher levels of objective hazard were also associated with higher probability of never missing a dose) (b = − 0.11, p = 0.1106).

### Momentary mental health

All momentary mental-health outcomes are plotted in Figs. 3, 4, and Figure S2. Positive mood had a robust association with subjective safety (b = 1.15, p < 0.0001), with the personal average level of positive mood steadily increasing across the range of personal average subjective safety values. Negative mood was generally low (as it has tended to be in all the populations we have studied previously. The lowest level of negative mood was the likeliest level across most of the range of subjective safety values, but the second lowest level of negative mood became most likely when subjective safety was low (subjective safety: b = − 1.14, p < 0.0001); none of the participants had a personal average negative mood above 4 on the 5-level Likert scale. There was a much weaker—and paradoxical—relationship between negative mood and objective hazard, with the probability of more-than-minimal negative mood being higher in participants with lower exposure to objective hazard (b = − 0.22, p = 0.0101). Self-reported stress was lower in participants with high levels of subjective safety (b = − 0.92, p < 0.0001), as expected, but was also slightly lower in participants with high levels of objective hazard (b = − 0.18, p = 0.012), another paradoxical effect.

### Correlations with the Perceived Neighborhood Scale

Prior to starting the EMA phase of the study, all participants were assessed with the PNS, referring to their home neighborhood. All four domains of the instrument were scored such that higher scores were worse. Objective hazard scores were positively correlated with two of the PNS domains: perceived crime (r = 0.18, p = 0.014) and dissatisfaction with the neighborhood (r = 0.18, p = 0.015). Subjective safety scores were negatively correlated with three of the PNS domains: perceived crime (r = − 0.25, p < 0.001), dissatisfaction with neighborhood (r = − 0.34, p < 0.001), and a lack of sense of community (r = − 0.3, p < 0.001). These correlations were all in the expected direction. Neither of our scores was substantially correlated with the PNS domain that assessed social embeddedness.

## Discussion

A recent systematic review [34] concluded that “neighborhood disadvantage, *regardless of whether it is assessed objectively or subjectively,* is one of the most robust correlates of HIV risk” (emphasis added). To our knowledge, the present study is the first to simultaneously track HIV transmission risk behavior along with objective and subjective qualities of the current neighborhood. Contrary to our expectations, we did not find that transmission risk was higher in participants who had more exposure to neighborhood-level disorder as indexed objectively. On average, the neighborhoods represented in our sample had higher objective hazard levels compared to the Baltimore average, but they still covered a wide range. Also on average, most participants felt subjectively safe most of the time; based on our previous studies with different populations, we suspect that this is true for most people, and it does not imply that our sample did not include a wide enough range of people. As mentioned above, we believe that many or most people find a niche where they feel safe, even if they live in an objectively disordered neighborhood.

Only one type of risky behavior was clearly associated with neighborhood-level objective hazard, and not as expected: having sex for money, drugs, or things (or having sex with a partner who does so) was *less* likely in participants with higher exposure to objective hazard. This finding did not seem to be straightforwardly associated with demographics (race, gender, HIV status), because they did not differ between the subsets who engaged in these risky activities and the sample as a whole.

In contrast with the lack of evidence for a relationship between transmission risk and objective hazard, there was clear evidence that subjective safety was associated with less of each type of transmission risk we studied except for sex without a condom. Overall, about 25% of participants who had sex did so at least once without a condom (Fig. 2), and this behavior was not substantially related to objective hazard or subjective safety. Each of the other five risky behaviors (needle sharing, sex with a casual partner, transactional sex, and sex with a partner who has transactional sex) was substantially less likely in participants who reported high levels of subjective safety (Fig. 2).

These findings suggests that people in subjectively safe environments might be more careful about transmission risk *because* they feel safe—reflecting either their having more cognitive energy to expend on considerations of personal health-related behaviors, or their feeling more hopeful and invested in their own futures and those of their companions. The latter explanation would be broadly consistent with findings in other city dwellers that expectations of early death are associated with riskier behavior [35] and worse socioeconomic outcomes [36]. It is also possible that some participants felt less safe because they were trapped in situations where they were compelled to engage in risky sexual or drug-use behavior. Conditions such as substance use disorder or hypersexuality might also lead people to inhabit unsafe places and to engage in risky behavior there.

Our most robust findings were that subjective safety had clear associations with antiretroviral therapy compliance, positive mood, negative mood, and stress: participants with higher levels of subjective safety reported better values for each of these outcomes. In a previous study with a nationwide sample of United States residents, most of whom were white, Robinette et al. [37] found that people who perceived their neighborhood as less safe had higher negative affect than others, especially in reaction to a stressful event. Those findings are consistent with our findings here (looking at stress and negative mood in a sample of predominantly black HIV+ participants), and they indirectly support the hypothesis that a lack of subjective safety, combined with stressful events and negative mood, might predict or even cause HIV transmission-risk behavior [22–25].

Our modified-snowball recruiting procedure—in which HIV− participants were enrolled only if they were invited by an HIV+ participant—was effective in ensuring that each HIV− participant was in the social circle of an HIV+ participant, but it also led to our sample having fewer HIV− participants than HIV+ participants. We do not know how well our findings would generalize to other geographical regions or to a random sample of participants within the same city. However, as has been noted previously, “HIV is not randomly distributed in neighborhoods, but instead concentrated in neighborhoods characterized by factors such as high rates of poverty, crime, and abandoned buildings.” [34]. This suggests that, given the existing systemic disparities, something like a snowball recruiting procedure (despite its disadvantages) is exactly what is needed to obtain the information we sought in this study.

Like all activity-space data, our data show associations, but not causal effects. Causal conclusions could be best assessed with random assignment to places, an approach that has only occasionally been achieved, either through residential-relocation projects [38], residence-improvement projects with designated control residences [39], or experimental allocation to momentary locations [40] or momentary forms of social interaction [41]. If the associations we observed here are shown to be modifiable risk or protective factors, then it is likely that practical applications can be developed to use simpler, more widely deployable screening tools that do not rely on the intensive collection of momentary geolocation data used here. For example, our objective hazard scores were based on the neighborhoods that participants were in when they were randomly prompted, but similar information could be obtained more quickly by asking participants to identify the neighborhoods where they spend time. Our subjective safety scores were derived from real-time assessments using EMA items that were adapted from the Perceived Neighborhood Scale [30], and some of these EMA scores were correlated with PNS domains, suggesting that the PNS and other brief questionnaires could be useful for identifying individuals or neighborhoods that might benefit from interventions designed to decrease HIV transmission risk and to increase psychosocial well-being.

## Supplementary Information

Below is the link to the electronic supplementary material.

Supplementary file1 (PDF 249 KB)
