# Supplementary material for: Objective Neighborhood-Level Disorder Versus Subjective Safety as Predictors of HIV Transmission Risk and Momentary Well-Being
Source: AIDS Behav. 2024 Jul 4;28(10):3326–37. doi: 10.1007/s10461-024-04413-z (PMC11427584; doi:10.1007/s10461-024-04413-z)
Supplement: Supplementary file 1 — Supplementary file1 (PDF 249 KB) [file 10461_2024_4413_MOESM1_ESM.pdf]

Supplementary figures and table for:

Objective neighborhood-level disorder versus subjective safety ratings as predictors of HIV transmission risk and momentary well-being

Figure S1. Tree diagram showing the types of networks obtained through the modified-snowball recruiting procedure, in which HIV+ participants recruited participants who could be either HIV+ or HIV-. "Plus" signs in circles: HIV+ participants. "Minus" signs in squares: HIV- participants. Participants who were not in a network within the study (singletons) are not shown. See main Results text for further details.

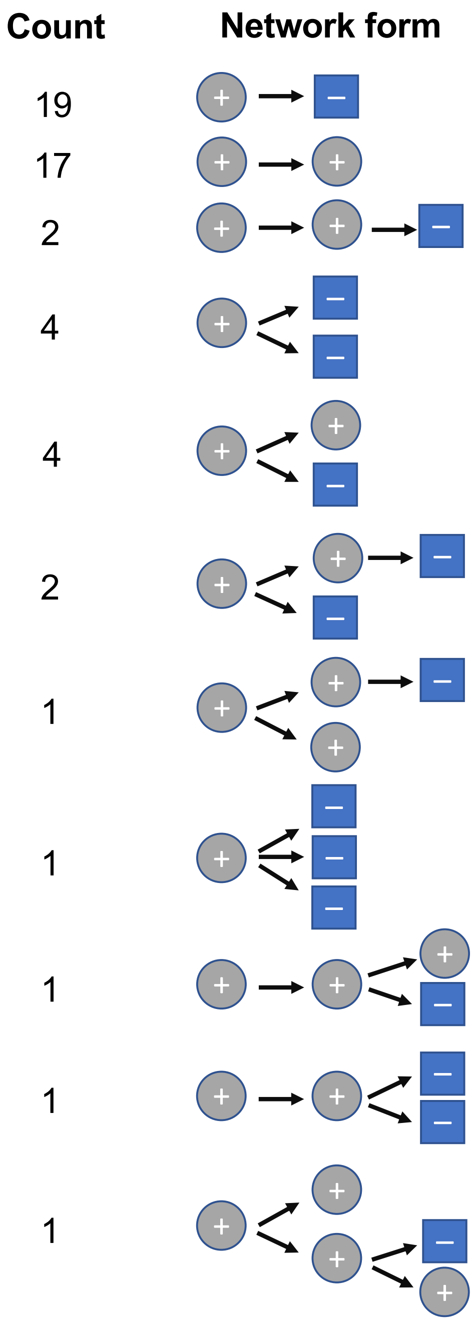

Figure S2. Illustration of the relationship between the regressors (objective hazard and subjective safety) and each ordinal outcome, from the same models shown in Figs. 3 and 4. This figure shows the probability of each level of each ordinal outcome, as a function of objective hazard and subjective safety. Lines show the median probability, and confidence bands show the 90% credible intervals.

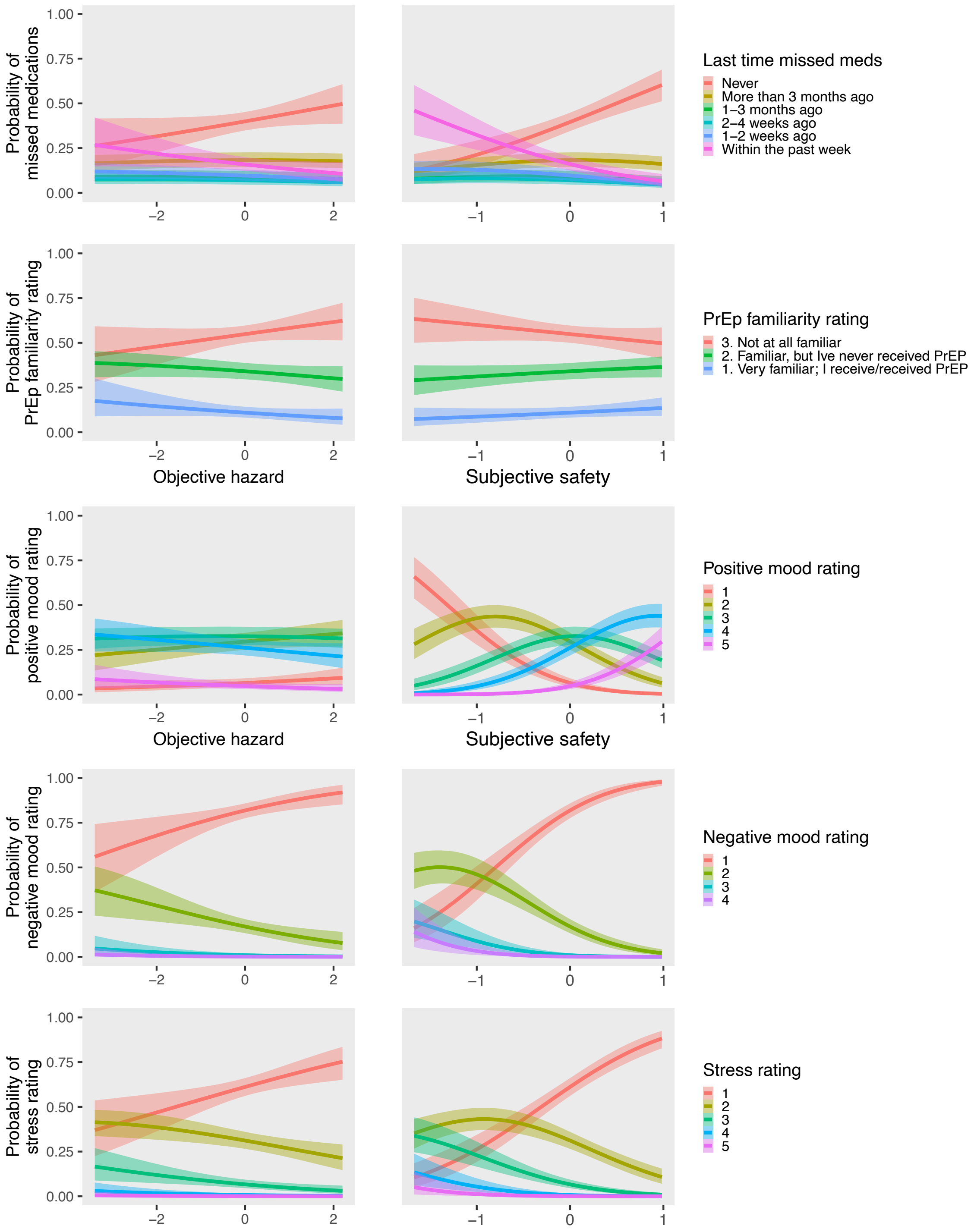

Table S1. Coefficient values for all statistical models. Binary outcomes were modeled using the Bernoulli family. Ordinal outcomes were modeled using the cumulative probit family. Abbreviations: N = number of participants; Coef = mean value of the model coefficient; SD = standard deviation of the model coefficient; CI_low = lower limit of 95% credible interval; CI_high = upper limit of 95% credible interval; Bayes p = probability that the posterior distribution of the coefficient contains zero.

Binary Outcome	N	Parameter	Coef	SD	CI_low	CI_high	Bayes p	
Did Not Use Condom	167	Intercept	-1.13	0.18	-1.44	-0.84	<0.0001	***
		Objective hazard	0.22	0.18	-0.08	0.52	0.2216	
		Subjective safety	0.26	0.3	-0.22	0.76	0.371	
Sex with Casual Partner	168	Intercept	0.08	0.16	-0.19	0.33	0.6322	
		Objective hazard	0.13	0.15	-0.13	0.38	0.4133	
		Subjective safety	-0.46	0.26	-0.87	-0.02	0.072	(*)
Had sex for money	168	Intercept	-0.55	0.17	-0.82	-0.26	<0.0001	***
		Objective hazard	-0.38	0.17	-0.66	-0.11	0.0212	*
		Subjective safety	-0.88	0.28	-1.33	-0.42	<0.0001	***
Partner injects drugs	167	Intercept	-0.9	0.18	-1.19	-0.61	<0.0001	***
		Objective hazard	0.05	0.18	-0.24	0.34	0.7803	
		Subjective safety	-0.79	0.28	-1.25	-0.33	0.0051	**
Partner has sex for money	166	Intercept	-0.86	0.18	-1.15	-0.57	<0.0001	***
		Objective hazard	-0.28	0.17	-0.56	0.01	0.1108	
		Subjective safety	-0.77	0.28	-1.23	-0.31	0.0045	**
Works used by another	42	Intercept	-0.21	0.39	-0.84	0.42	0.5883	
		Objective hazard	0.16	0.32	-0.38	0.69	0.6256	
		Subjective safety	-0.97	0.55	-1.86	-0.04	0.0709	(*)

Ordinal outcome	N	Parameter	Coef	SD	CI_low	CI_high	Bayes p	
Time since missed medication	247	Threshold[1]	-0.27	0.08	-0.4	-0.14	0.0011	**
		Threshold[2]	0.2	0.08	0.06	0.33	0.0158	*
		Threshold[3]	0.43	0.08	0.29	0.57	<0.0001	***
		Threshold[4]	0.64	0.09	0.5	0.79	<0.0001	***
		Threshold[5]	0.99	0.1	0.83	1.15	<0.0001	***
		Objective hazard	-0.11	0.07	-0.23	0	0.1106	
		Subjective safety	-0.53	0.12	-0.74	-0.33	0	***
Familiarity with PrEP	286	Threshold[1]	0.12	0.07	0	0.24	0.101	
		Threshold[2]	1.23	0.1	1.06	1.39	<0.0001	***
		Objective hazard	-0.09	0.07	-0.2	0.03	0.2107	
		Subjective safety	0.13	0.11	-0.06	0.32	0.2549	
Positive mood	286	Threshold[1]	-1.52	0.11	-1.7	-1.33	<0.0001	***
		Threshold[2]	-0.35	0.08	-0.49	-0.22	<0.0001	***
		Threshold[3]	0.5	0.08	0.36	0.63	<0.0001	***
		Threshold[4]	1.67	0.12	1.47	1.87	<0.0001	***
		Objective hazard	-0.09	0.06	-0.2	0.01	0.1551	
		Subjective safety	1.15	0.12	0.96	1.35	<0.0001	***
Negative mood	286	Threshold[1]	0.91	0.1	0.76	1.07	<0.0001	***
		Threshold[2]	2.29	0.19	1.98	2.6	<0.0001	***
		Threshold[3]	2.99	0.28	2.54	3.45	<0.0001	***
		Objective hazard	-0.22	0.09	-0.36	-0.08	0.0101	*
		Subjective safety	-1.14	0.15	-1.39	-0.9	<0.0001	***
Stress	286	Threshold[1]	0.28	0.08	0.15	0.41	<0.0001	***
		Threshold[2]	1.44	0.11	1.26	1.62	<0.0001	***
		Threshold[3]	2.4	0.2	2.08	2.72	<0.0001	***
		Threshold[4]	3.2	0.36	2.61	3.76	<0.0001	***
		Objective hazard	-0.18	0.07	-0.3	-0.06	0.0121	*
		Subjective safety	-0.92	0.12	-1.12	-0.71	<0.0001	***
